# *Escherichia coli* in Iran: An Overview of Antibiotic Resistance: A Review Article

**Published:** 2018-01

**Authors:** Hesam ALIZADE

**Affiliations:** Infectious and Tropical Diseases Research Center, Hormozgan Health Institute, Hormozgan University of Medical Sciences, Bandar Abbas, Iran

**Keywords:** *Escherichia coli*, Antibiotic resistance, *E. coli* infections

## Abstract

**Background:**

*Escherichia coli* is the most prominent cause of infectious diseases that span from the gastrointestinal tract to extra-intestinal sites such as urinary tract infection, septicaemia, and neonatal meningitis. The emergence and spread of antibiotic resistance in *E. coli* is an increasing public health concern across the world. Rising resistance in *E. coli* isolates is also observed in Iran. This review summarizes the status of antibiotic resistance of *E. coli* isolates in Iran from 2007 to 2016.

**Methods:**

The data of the prevalence of *E. coli* antibiotic resistance were collected from databases such as Web of Science, PubMed, Scopus, Embase, Cochrane Library, Google Scholar and Scientific Information Database.

**Results:**

Antibiotic resistance in *E. coli* is on the rise.

**Conclusion:**

Prevalence of antibiotic resistance of *E. coli* varies from region to region in Iran.

## Introduction

Over the past decade increasing antibiotic resistance among isolates of Enterobacteriaceae has become a main public health concern (1). In the most recent estimates of global antibiotic resistance published by the WHO in 2014, *Escherichia coli* was named as one of the biggest concerns associated with hospital and community-acquired infections (2).

Pathogenic *E. coli* is one of the major causes of infectious diseases that span from the gastrointestinal tract to extra-intestinal sites such as the urinary tract, bloodstream, and central nervous system (3,4).

*E. coli* is the most common producers of Extended-Spectrum Beta-Lactamases (ESBLs) (5). The presence of ESBLs enzymes compromises the efficacy of all β-lactams, excepting cephamycins and carbapenems, by hydrolysis of the β-lactam ring, and play a major role in the inhibition of the penicillin-binding protein targets (6). More than 300 different ESBL enzymes have been recognized so far (7). Since the early 2000s, CTX-M enzymes have been increasingly detected, and these enzymes have now replaced other ESBLs such as TEM and SHV as the most common type of ESBL (6, 8). Other enzymes having ESBL have also been described (e.g. PER, VEB-1, BES-1, CME-1, SFO-1, and GES-1) (9).

Due to the rising percentage of bacteria-carrying ESBL genes, there has been a corresponding increase in the clinical use of antibiotics of the carbapenem group. The hallmark of carbapenemases enzymes is its ability to inactivate carbapenems and extended-spectrum cephalosporins (10).

Metallo-β-lactamase (MBLs) enzymes are now widespread and found in Asia, Europe, Canada, Australia, and South and North America (11). The fluoroquinolones are potent antibiotic agents used in the prophylaxis and treatment of infections caused by *E. coli*. Fluoroquinolone-resistant *E. coli* strains often indicate resistance to all main classes of available antimicrobials such as gentamicin, tetracycline, ampicillin, chloramphenicol, and trimethoprim/sulfamethoxazole (12). The aminoglycosides are powerful bactericidal agents often used along with a spectrum beta-lactams. Resistance to aminoglycosides is most commonly caused by aminoglycoside modifying enzymes such as phosphorylate (aminoglycoside phosphoryl transferase [APH]), acetylate (aminoglycoside acetyltransferase [AAC]) or adenylate (aminoglycoside nucleotidyltransferase [ANT]) (13).

The genes encoding resistance to sulfonamide-class antibiotics such as *sul1*, *sul2*, and *sul3*, which competitively inhibit dihydropteroate synthetase activity, are highly prevalent among Gram-negative bacteria isolated from human samples (14). Unfortunately, the *sul* genes have the highest prevalence in *E. coli* isolates (14, 15).

Trimethoprim (TMP) inhibits dihydrofolate reductase that catalyses the formation of tetrahydrofolate from dihydrofolate. The most prevalent of the *dhfr* genes, *dhfrI* and variants of *dhfrII*, mediate high-level resistance to TMP and are most frequently found in Gram-negative enteric bacteria (16).

The purpose of this review was assessing the exact magnitude of *E. coli* antibiotic resistance in peer-reviewed published literature in Iran over the last nine years.

## Methods

### Literature search strategy

From 2007 to 2016, all published literature addressing antibiotic resistance of *E. coli* in Iran were collected from databases Web of Science, PubMed, Scopus, Embase, Cochrane Library, Google Scholar and Scientific Information Database. The following keywords containing Medical Subject Headings or keywords in titles or abstracts were used “*E. coli*” [MeSH] AND “antibiotic resistance” [MeSH] AND “Iran” [MeSH].

### Inclusion and exclusion criteria

All original articles that presented cross-sectional or cohort studies and reported the prevalence of antibiotic resistance of *E. coli* in Iran were considered.

### Data analysis

The analysis for the descriptive data was carried out using SPSS software (Chicago, IL, USA, ver. 19).

## Results and Discussion

### Epidemiology of antibiotic resistance

In 2015 the Eastern Mediterranean regional office of WHO reported that none of the participating countries had a national action plan for antimicrobial resistance, considered a priority and an outcome indicator for control measures (17). In Iran, like other Eastern Mediterranean countries, antibiotics can easily be obtained over the counter. Antimicrobial medicines are often prescribed at the request of patients, and pharmacies do not necessarily comply with regulations. Many people in the Eastern Mediterranean region believe that antibiotics help in most ailments with fever. Poor-quality and counterfeit antimicrobial medicines are a particular problem with respect to antimicrobial resistance in these regions (17–19).

In Iran antibiotic resistance in Gram-negative bacteria is on the rise, particularly in *E. coli* (20–23). Different patterns of antibiotic resistance is seen in various regions across the Iran: For example, more than 90% of *E. coli* isolates were resistant to penicillin (ampicillin or amoxicillin) in Tehran (capital) (24, 25) (Table 1).

**Table 1:** Antibiotic resistance pattern of *E. coli* strains isolated from human sources based on disk diffusion method in various regions of Iran

***City***	***Source***	***AMG***	***PCN***	***CEPH***	***FLQ***	***MAC***	***IMP***	***SXT***	***TET***	***CAM***	***NAL***	***ESBL***	***Ref***
Babol (north)	Urine	36.80	-	45.60	24.60	-	38.60	64.90	-	-	-	-	(71)
Rasht (north)	Urine	59.9	68.2	41.8	43.6	-		60	60	-	47.3	-	(29)
Rasht (north)	Urine, MDR	33.33	-	60.60	36.36	-	36.36	-	78.78	45.45	-	24.00	(30)
Rasht (north)	Urine	36.36	-	51.51	33.33	-	33.33	-	81.81	45.45	-	24.00	(31)
Karaj (north)	UTI	73.69	73.69	38.16	26.32	9.22	15.79	69.74	-	-	60.53	-	(47)
Tabriz (north west)	Clinical sample	45.70	99.30	46.40	47.60	12.90	1.40	75.00	72.80	20.70	60.70	-	(26)
Zanjan (north west)	Clinical sample	28.50	68.50	31.50	52.20	-	0.00	46.50	-	-	-	33.00	(32)
Zanjan (north west)	EAEC, children	10.70	18.60	15.00	12.10	25.70	0.70	5.70	17.10	-	-	-	(44)
Zanjan (north west)	Stool, children	18.60	55.70	47.80	25.00	74.30	1.40	15.70	52.10	-	-	-	(44)
Tabriz (north west)	Clinical sample	67.90	-	63.30	40.80	-	6.30	61.90	-	-	54.90	66.20	(48)
Kermanshah (west)	UTI, ESBL	30.60	93.90	73.50	42.90	4.10	0.00	75.50	-	-	-	24.50	(28)
Sanandaj (west)	Diarrhea children	-	79.80	30.30	30.30	20.20	-	70.70	89.90	88.90	36.40	-	(72)
Sanandaj (west)	Clinical sample, MDR	49.00	-	68.60	64.70	47.00	47.00	88.20	29.40	-	56.80	-	(58)
Sanandaj (west)	Urine	45.03	84.97	32.54	19.97	19.97	10.03	75.02	89.89	86.00	75.02	19.02	(51)
Hamadan (west)	Diarrhea	35.00	-	85.00	32.50	10.00	-	50.00	-	-	62.50	-	(73)
Hamadan (west)	UTI	53.30	-	87.00	39.10	23.90	-	66.00	-	-	59.00	-	(73)
Hamedan (west)	Stool	27.50	87.50	75.00	5.00	-	-	72.50	75.00	35.00	22.50	-	(22)
Hamadan (west)	UTI, children	-	-	30.00	-	0.00	-	70.00	-	-	47.00	-	(61)
Hamadan (west)	UTI, children	17.50	33.30	35.00	15.00	0.00	2.50	70.80	-	-	40.90	27.3	(38)
Mashhad (north east)	Clinical sample	-	-	52.00	43.00	-	-	-	-	-	-	42.50	(60)
Semnan (north east)	Urine	25.20	99.10	28.30	40.20	-	-	63.40	67.70	-	54.90	29.20	(27)
Semnan (north east)	Clinical sample	27.60	98.50	18.90	25.80	-	-	58.20	53.50	20.00	-	17.45	(27)
Kashan (central)	Clinical sample	-	-	-	-	-	-	-	-	-	-	46.60	(74)
Kashan (central)	Clinical sample	38.80	76.10	30.60	21.60	-	0.00	-	-	-	-	-	(75)
Isfahan (central)	UTI	14.84	69.53	59.76	55.46	19.40	0.00	25.00	-	-	23.43	43.67	(59)
Isfahan (central)	UTI	-	-	34.00	39.00	6.00	-	29.00	-	-	63.00	-	(52)
Isfahan (central)	UTI	-	94.53	51.66	45.83	11.85	1.20	-	-	-	-	36.11	(76)
Arak (central)	Clinical sample	-	-	-	-	-	-	-	-	-	-	80.50	(34)
Kashan (central)	EPEC, children	43.10	100	39.20	35.30	-	0.00	-	-	-	62.70	-	(54)
Tehran (capital)	Stool, children	51.29	89.60	-	28.60	-	-	38.96	83.10	59.74	-	-	(42)
Tehran (capital)	UTI	-	100	2.60	-	-	-	-	-	-	-	2.40	(24)
Tehran (capital)	STEC	62.29	36.06	-	2.45	1.63	-	-	86.88	1.63	-	-	(50)
Tehran (capital)	UTI	17.07	36.58	-	19.51	5.69	-	-	73.98	25.20	-	-	(68)
Tehran (capital)	Clinical sample	36.20	91.50	39.50	39.00	94.00	0.00	57.00	58.50	-	-	70.00	(25)
Tehran (capital)	UTI	40.00	81.30	56.70	61.30	17.30	0.70	64.70	-	-	71.30	-	(53)
Tehran (capital)	UTI	-	28.00	69.30	19.33	-	-	-	-	-	-	28.00	(40)
Tehran (capital)	EPEC, children	0.00	5.70	2.80	1.40	-	-	4.20	18.50	2.80	1.40	-	(45)
Tehran (capital)	EPEC, children	-	61.90	19.00	16.70	-	0.00	54.80	38.10	2.38	-	21.40	(71)
Tehran & Ilam (capital & west)	UTI	-	81.25	40.97	-	3.47	-	60.41	58.33	-	-	50.00	(14)
Central, western & northern	Diarrhea	6.00	62.00	7.00	3.00	-	-	39.00	63.00	31.00	4.00	-	(57)
Jahrom (south)	Urine, children	15.60	80.20	10.40	8.30	3.10	0.00	76.00	70.80	35.40	25.00	-	(62)
Jahrom (south)	Urine	11.70	-	20.00	21.70	3.30	-	45.00	-	-	41.70	-	(77)
Shiraz (south)	Diarrhea	8.33	36.11	16.67	8.33	5.56	5.56	41.67	41.67	13.89	-	12.96	(41)
Yasouj (south west)	UTI	15.50	76.00	40.50	29.00	-	1.00	62.00	50.00	13.00	48.50	-	(78)
Kerman (south east)	Diarrhea	-	-	40.77	-	-	2.77	-	-	-	-	25.92	(39)
Kerman (south east)	Clinical sample	-	-	37.00	-	-	0.00	-	-	-	-	68.00	(35)
Kerman (south east)	Clinical sample	39.30	91.40	31.00	44.90	-	0.00	93.40	83.70	-	71.90	43.70	(65)
Kerman (south east)	Urine	36.45	-	-	29.18	6.25	0.00	60.42	-	-	54.16	-	(79)
Bam (south east)	Clinical sample	52.30	-	28.70	24.30	-	-	39.70	-	-	59.70	-	(80)
Zabol (south east)	UTI	-	-	-	-	-	-	-	-	-	-	44.40	(81)
Zabol (south east)	Cervico-vaginal	77.27	94.69	61.36	31.81	-	34.93	67.42	92.42	-	88.63	-	(46)
Zahedan (south east)	Urine	76.60	93.30	54.40	-	-	-	73.30	90.00	-	67.70	62.70	(43)

AMG: aminoglycoside, PCN: penicillin, CEPH: Third-generation cephalosporine, FLQ: fluoroquinolone, MAC: macrolide, IMP: imipenem, SXT: co-trimoxazole, TET: tetracycline, CAM: chloramphenicol, NAL: nalidixic acid, ESBL: extended spectrum beta-lactamase, MDR: multi-drug-resistant

The rate of resistance of *E. coli* isolates in four countries to third-generation cephalosporins was 22%–63% (2). Many studies conducted in Iran have also revealed a similar resistance rate of *E. coli* isolates to third-generation cephalosporins in various regions (26, 28–32) (Table 1). In Iran, cephalosporins are widely used because of their low rate of side effects. This may be related to the increased resistance to these antibiotics (33).

**Fig. 1:**
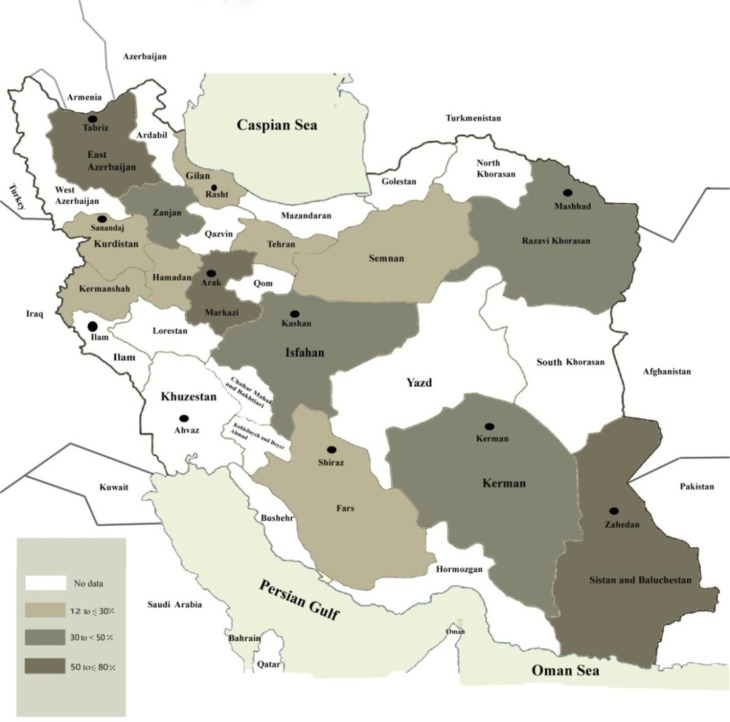
Prevalence of ESBLs-producing *E. coli* clinical isolates in Iran

β-lactamase enzymes production in *E. coli* is the most important mediator of resistance to a broad spectrum of β-lactams antibiotics (6, 7). Based on previous reports from various regions in Iran, high prevalence of ESBL phenotype of *E. coli* was detected in Arak (central) (34), Kerman (south-east) (35) and Tabriz (north-west) (36) (Table 1) (Fig. 1).

Among ESLB gene families, *bla_CTX-M_*, *bla_TEM_*, and *bla_SHV_* are the most common in *E. coli* ESBL-producing isolates obtained from various clinical samples (37). A high prevalence of *bla_CTX-M_* (66.70%) (38) and *bla_CTX-M-1_* (61.08%) (25) were detected in *E. coli* isolates. Moreover, *bla_CTX-M-1_* and *bla_CTX-M-15_* were present in 95.30% (34) and 91.07% (39) *E. coli* ESBL-producing isolates. *bla_TEM_* gene was reported in 95.20% (40) and 83.33% (41) of *E. coli* isolates from Tehran (capital) and Shiraz (south), respectively. Gene carriage for ampicillin resistance gene (CITM) was observed in 90.25% (highest distribution) (42) and 5.00% (lowest distribution) (43) of the *E. coli* isolates.

Unfortunately, there is very little data concerning carbapenem resistance of *E. coli* isolates in Iran. The presence of *bla_IMP_*, *bla_VIM_*, and *bla_NDM-1_* genes from EAEC isolates in children were reported and none of the isolates possessed these genes (44).

None of the ESBL-producing *E. coli* isolates were positive for *bla_IMP_* and *bla_VIM_* in Kerman, southeastern Iran (39). The distribution of resistance genes among *E. coli* isolates is summarized in Table 2.

**Table 2:** Prevalence of antibiotic resistance genes in *E. coli* strains (percentage) isolated from human source in Iran

***Antimicrobial agent***	***Target gene***	***Ref***
Beta-lactams	*bla_TEM_* (19.00), *bla_SHV_* (40.50), *bla_CTX-M-1_* (19.04), *bla_CTX-M-2_* (0.00), *bla_CTX-M-9_* (0.00), *bla_CTX-M-15_* (19.04)	(82)
	*bla_TEM_* (49.00), *bla_SHV_* (44.00), *bla_CTX-M_* (28.00), *bla_VEB_* (8.00), *bla_GES_* (0.00)	(63)
	*bla_CTX-M-1_* (95.30), *bla_CTX-M-2_* (35.10), *bla_CTX-M-8_* (16.60), *bla_CTX-M-9_* (45.30)	(34)
	*bla_TEM_* (85.20), *bla_SHV_* (53.20), *bla_CTX-M_* (26.10)	(14)
	*bla_TEM_* (83.33), *bla_SHV_* (31.48), *bla_CTX-M_* (20.37)	(41)
	*bla_TEM_* (43.50), *bla_SHV_* (34.80), *bla_CTX-M_* (15.90)	(32)
	*bla_TEM_* (40.80), *bla_SHV_* (20.80), *bla_CTX-M_* (66.70)	(35)
	*bla_CTX-M-15_* (91.07), *bla_OXA-1_* (1.78), *bla*_*PER*-1_ (0.00)	(39)
	*bla_CTX-M-1_* (61.08), *bla_CTX-M-2_* (0.00), *bla_CTX-M-9_* (0.00)	(20)
	*bla_TEM_* (49.10), *CITM* (5.00), *blafox* (0.00)	(25)
	*bla_TEM_* (46.96), *bla_SHV_* (56.00)	(32)
	*bla_TEM_* (12.14), *bla_SHV_* (7.47)	(59)
	*bla_SHV_* (27.64), *CITM* (39.83)	(68)
	*bla_SHV_* (56.55), *CITM* (48.36)	(50)
	*bla_SHV_* (57.79), *CITM* (90.25)	(42)
	*bla_TEM_* (76.47), *bla_SHV_* (27.00)	(60)
	*bla_TEM_* (60.00), *bla_SHV_* (26.00)	(24)
	*bla_TEM_* (63.00), *bla_SHV_* (7.00)	(75)
	*bla_TEM_* (95.20), *bla_SHV_* (26.10)	(40)
	*bla_CTX-M_* (14.70)	(65)
	*CITM* (38.59)	(67)
	*AmpC* (24.00)	(24)
	*bla_IMP_* (0.00), *bla_VIM_* (0.00), *bla_NDM-1_* (0.00)	(44)
	*bla_IMP_* (0.00), *bla_VIM_* (0.00)	(42)
Aminoglycoside	*aac (3)-IIa* (78.87), *ant(2)-Ia* (47.88)	(49)
	*aadA1* (52.84), *aac(3)-IV* (22.76)	(68)
	*aadA1* (60.65), *aac(3)-IV* (68.03)	(50)
	*aadA1* (96.10), *aac(3)-IV* (54.54)	(42)
Quinolone	*qnrA* (31.50), *qnrB* (17.00)*, qnrS* (7.00)	(60)
	*qnrA* (37.50), *qnrB* (20.80)*, qnrS* (0.00)	(40)
	*qnrA* (0.00), *qnrB* (6.66)*, qnrS* (5.00)	(38)
	*qnr* (46.34)	(68)
	*qnr* (12.29)	(50)
	*qnr* (72.07)	(42)
	*qnr* (15.78)	(67)
Tetracycline	*tetA* (85.06), *tetB* (84.41)	(42)
	*tetA* (43.80), *tetB* (36.58)	(68)
	*tetA* (51.63), *tetB* (38.52)	(50)
	*tetA* (10.52)	(67)
Chloramphenicol	*cat1* (59.74), *cmlA* (60.38)	(42)
	*cat1* (15.44), *cmlA* (15.44)	(68)
	*cat1* (0.81), *cmlA* (0.81)	(50)
Co-trimoxazole	*sul1*(81.60), *sul2* (66.70), *sul3* (2.30), *dfrA1* (39.10), *dfrA5* (5.70)	(14)
	*dfrA1* (51.94), *Sul1* (40.25)	(42)
	*dfrA1* (63.15), *Sul1* (17.54)	(67)
	*dfrA1* (21.95), *Sul1* (36.58)	(68)
	*dfrA1* (36.06), *Sul1* (82.78)	(50)
Integrons	*IntI*1 (22.03), *IntI*2 (5.08), *IntI*3 (0.00)	(26)
	*IntI*1 (6.25), *IntI*2 (10.41), *IntI*3 (0.00)	(62)
	*IntI*1 (78.26), *IntI*2 (76.81), *IntI*3 (26.09)	(83)
	*IntI*1 (52.00), *IntI*2 (2.50), *IntI*3 (0.00)	(78)
	*IntI*1 (47.05), *IntI*2 (3.92)	(58)
	*IntI*1 (78.20)	(14)
	*IntI*1 (97.00)	(31)

The prevalence of isolates resistant to aminoglycosides ranged from 0.00% among EPEC isolated from children (Tehran, capital) (45) to 77.27% among *E. coli* isolated from Cervico-vaginal (Zabol, south-eastern Iran) (46). The percentage is also higher in Zahedan (south-east) (43), Karaj (north) (47), and Tabriz (north-west) (48). Among aminoglycoside-modifying enzymes, resistance against gentamicin, kanamycin, cidomycin, and tobramycin in *E. coli* is mediated by ANT (2″)-Ia enzyme, coded by *ant(2″)-Ia* gene. *aac (6′)-Ib* gene is more common and leads resistance to kanamycin, tobramycin, and amikacin; Simultaneous resistance to gentamycin and tobramycin mediated by AAC(3)-IIa enzyme coded by aac(3)-IIa gene (49). The prevalence of different resistance genes varied—96.10% for the *aadA1* gene (42), 68.03% for the *aac(3)-IV* gene (50), 78.87% for the *aac(3)-IIa* gene, and 47.88% for the ant(2)-Ia gene (49).

Nalidixic acid is an antibiotic from the first generation of quinolones. Nowadays resistance to this antibiotic has increased substantially across Iran (26, 43, 46, 47, 51–54).

Fluoroquinolones are highly efficacious antimicrobial agents, often preferred as initial agents for empirical therapy of UTIs. Unfortunately, urinary tract *E. coli* isolates in both hospitalized and out-patients are becoming increasingly resistant to commonly used fluoroquinolones (55, 56). The prevalence of fluoroquinolone-resistant isolates ranged from about 1%–3% (45, 50, 57) to more than 50% in Iran (32, 55, 58, 59). *qnr* genes (*qnrA*, *qnrB*, and *qnrS*) may facilitate the spread and increase the prevalence of quinolone-resistant strains. To date, *qnr* genes have been widely identified in Southern and Eastern Asia (82, 60). In earlier studies in Iran, the most prevalent gene among all isolates was *qnrA*, followed by *qnrB* and *qnrS* (40, 60). *qnrS* has been reported previously from clinical isolates of *E. coli* in Mashhad (60) and has also been detected in UTI isolated from children *E. coli* isolates from Hamadan (38). Our pooled evidence showed that the prevalence of macrolide resistance among *E. coli* clinical isolates varied from 0%–3% in Tehran (sample source: STEC), Hamadan (UTI from children), and Jahrom (urine from children) to 94% in Tehran (various clinical samples) (25, 50, 61, 62) (Table 1).

In a study in Tehran, 39% of *E. coli* isolates were resistant to aztreonam (25). Resistance against aztreonam may be related to the production of ESBL enzymes by ESBL-producing strains (53). Uropathogenic *E. coli* strains showed high sensitivity to nitrofurantoin (47, 50, 53). Susceptibility to nitrofurantoin may result from decreasing the use of this drug in Iran (53).

The rate of colistin-resistant ESBL-Producing *E. coli* with the MIC test was 82% (63). Increasing use of colistin for treatment of various infections due Gram-negative bacteria has led to the emergence of colistin resistance in several countries Asia (especially Korea and Singapore) (64).

Percentages of *E. coli* isolates resistant to cotrimoxazole vary with the geographical location of the patients: 93.40% in Kerman (65) and 4.20% in Tehran (45). Among clinical *E. coli* isolates resistance to TMP varies greatly, ranging from 10% to 70% depending on geographical locations (66). A high prevalence of clinical resistance to TMP (*dfrA1* gene) was reported in enteric bacteria (14, 42, 50, 67). Only one city (Tehran) reported a decreasing trend (21.95%) (68).

Resistance to sulfonamide was one of the most common resistances detected by previous studies and is often associated with the acquisition of the resistance genes *sul1* and *sul2* (14, 50).

High prevalence of tetracycline resistance has been observed in *E. coli* isolated from human and animals around the world (69). Prevalence of *tetA* is higher than *tetB* gene in *E. coli* strains isolated from clinical samples (42, 50, 68).

The most developed countries have sufficient control of over-the-counter sales, while many drugs, including antibiotics, are easily available in many developing countries. In Iran, as in other developing countries, almost any antibiotic can be acquired over the counter without a prescription (19). In other cases, doctors might not advise laboratory tests to confirm bacterial infection and hence the antibiotic might be unnecessarily prescribed (70).

## Conclusion

Over the years, antimicrobial resistance in Iran has increased markedly in Gram-negative bacteria such as *E. coli*. This prevalence of antibiotic resistance of *E. coli* varies from region to region in Iran. However, it cannot fully represent the prevalence of antibiotic resistance of *E. coli* in Iran, because the extent of resistance to different antibiotic categories is yet to be examined in many areas of the country.

## Ethical considerations

Ethical issues (Including plagiarism, informed consent, misconduct, data fabrication and/or falsification, double publication and/or submission, redundancy, etc.) have been completely observed by the author.
